# Prenatal diagnosis of fetuses with renal abnormalities: a retrospective analysis of 329 Chinese cases

**DOI:** 10.1186/s13023-025-04001-x

**Published:** 2025-09-24

**Authors:** Yayun Qin, Bo Wang, Yuanyuan Zhu, Lijun Liu, Nian Liu, Yanyi Yao, Hui Li, Runhong Xu, Chengcheng Zhang, Jieping Song

**Affiliations:** 1 Medical Genetics Center, Maternal and Child Health Hospital of Hubei Province, Wuhan, 430070 Hubei Province China; 2 Berry Genomics Corporation, Beijing, 102200 China

**Keywords:** Renal anomalies, Chromosomal abnormalities, Whole-exome sequencing, Prenatal diagnosis, Optical genome mapping

## Abstract

**Background:**

There is no clear guidance on prenatal diagnostic testing strategies for congenital renal anomalies. Therefore, this study aims to investigate the retrospective analysis of ultrasound and genetic diagnostic results in cases of fetal renal abnormalities and to establish genotype-phenotype correlations.

**Methods:**

A total of 329 fetuses with renal abnormalities that underwent prenatal diagnostic testing from January 2020 to April 2023 were recruited in this study. These cases were classified into 11 subgroups based on their ultrasound diagnosis. All cases underwent chromosomal microarray analysis (CMA) or copy number variation sequencing (CNV-seq), with subsequent whole exome sequencing (WES) conducted on select CMA/CNV-seq negative cases, subject to parental consent for further testing targeting monogenic variations.

**Results:**

Of the 329 cases analyzed, CMA/CNV-seq detected chromosomal abnormalities in 31 cases, with a detection rate of 9.4% (31/329). The most common abnormality was 17q12 deletion, accounting for 29% of the positive cases (9/31) and 2.7% of the total cases (9/329). WES was conducted on 76 cases (76/298, 25.5%), revealing 16 monogenic variants, and 2 CNVs in 12 cases (15.8%). An overall positive diagnostic yield of 13.1% (43/329) was obtained in the pipeline of combinational CMA/CNV-seq and WES analysis. Ciliary genes (*TMEM67*, *NPHP3*, *CEP290*, *BBS2*, and *TTC8*) were frequently implicated by WES. Several genotype-phenotype correlations emerged, including (1) hyperechogenic kidneys associated with 17q12 deletion, (2) renal dysplasia, renal cysts, hydronephrosis, ectopic kidney, and renal duplication with chromosomal abnormalities, (3) unilateral renal agenesis and polycystic kidneys with monogenic variants.

**Conclusion:**

This study reveals genotype-phenotype correlations in fetal renal abnormalities, informing prenatal counseling regarding diagnostic testing options and expected outcomes.

**Supplementary Information:**

The online version contains supplementary material available at 10.1186/s13023-025-04001-x.

## Background

Fetal renal anomalies detected through prenatal ultrasound account for approximately 20% of birth defects based on 30,940 infants and fetuses from the Mainz congenital birth defect monitoring system (1990–1998) [1]. These anomalies can involve changes in kidney size, shape, position, or number. They encompass conditions such as renal agenesis, renal dysplasia, multicystic dysplastic kidney (MCDK), horseshoe kidney, ectopic/pelvic kidney, and others [2]. Additionally, they can affect the micro and macro composition of the kidney. The severity of these abnormalities can vary from asymptomatic incidental findings to abnormalities associated with end-stage renal disease.

These renal abnormalities, whether isolated or non-isolated, have diverse causes, including known or unknown environmental teratogenic factors and genetic disorders (chromosomal, monogenic, or polygenic) [3]. Certain loci, such as 1q21, 4p16.1-p16.3, 16p11.2, 16p13.11, 17q12, and 22q11.2, are significant “hotspots” for copy number variants (CNVs) associated with renal anomalies [4]. It is important to perform prenatal diagnosis to uncover the causes of fetal renal abnormalities. This will help to assess fetal prognosis, genetic counselling and clinical management. Prenatal chromosome microarray analysis (CMA) is a common first-tier diagnostic tool for fetal kidney disease, with diagnostic yields ranging from 2.44%-13.8% [5–8]. Additionally, copy number variant sequencing (CNV-seq) is used in prenatal diagnosis to detect CNVs [9, 10]. Antenatal whole exome sequencing (WES) effectively complements this process by identifying genetic causes of unexplained renal abnormalities when CMA results are negative. This adds a diagnosis in 7.32–19.38% of cases [6, 11, 12]. Therefore, CMA/CNV-seq and WES are valuable tools for detecting chromosomal aberrations (such as chromosomal aneuploidies and CNVs) and pathogenic sequence variants in fetal renal anomaly cases.

17q12 microdeletion syndrome, a rare genetic disorder caused by microdeletions in the long arm of chromosome 17, has a penetrance of approximately 34.4% [13]. Prenatal ultrasound typically reveals hyperechogenic kidneys or altered renal volume in 17q12 deletion cases. *De novo* 17q12 deletions account for approximately 55.6%, significantly more common than 17q12 duplications [13]. Haploinsufficiency of the *HNF1B* gene, primarily affected by 17q12 deletion, causes reduced renal function and diabetes [14, 15].

Although many prenatal diagnostic techniques are currently available, strict genotype-phenotype correlation is important for the analysis and interpretation of genetic data in the clinical setting. Establishing genotype-phenotype correlations can also help predict the clinical course of renal anomalies, guide treatment decisions, and improve prognostic accuracy. However, clear guidance for prenatal diagnostic testing strategies related to fetal renal anomalies, particularly regarding genotype-phenotype correlations, is lacking. Therefore, this study aimed to retrospectively analyze 329 fetuses with renal anomalies detected through ultrasound. The detection rates of CMA/CNV-seq and WES in fetuses with different clinical subtypes of renal anomalies were assessed. Genotype-phenotype correlations have been identified by correlating genetic causes with fetal clinical phenotypes. This study could significantly enhance the evaluation of CMA/CNV-seq and WES in specific prenatal kidney anomalies, improving prenatal diagnosis, genetic counseling, and clinical management.

## Methods

### Participant recruitment and DNA preparation

The 329 unrelated participants in this study were initially diagnosed with renal abnormalities during the antenatal ultrasound unit. Subsequently, clinical geneticists assessed the cases and provided recommendations to affected couples. Those consenting to genetic counseling and prenatal diagnostic testing had their samples, such as amniotic fluid or blood, collected and sent to the Medical Genetics Center for DNA extraction and genetic testing. The testing process started with CMA/CNV-seq. WES was offered if CNA/CNV-seq yielded negative results. This study recruited a total of 329 unrelated fetuses with renal abnormalities who underwent prenatal diagnostic testing from January 2020 to April 2023. The gestating women primarily presented with: [1] abnormal prenatal ultrasound findings (100%, referral indication) [2], advanced maternal age (≥ 35 years; 13.7%) [3], abnormal serum screening results (12.2%), or [4] renal anomaly history (8.5%). For those with renal anomalies, the maternal age ranged from 18 to 46 years, and the mean gestational age at diagnosis ranged from 17 to 34 weeks. All participants underwent ultrasound screening by two senior sonographers with extensive experience in prenatal ultrasound screening. The fetal ultrasound renal anomalies included in this study included renal dysplasia (including small kidney size), renal cysts, hydronephrosis (including pyelectasis, collection system separation, and hydroureter), MCDK, renal duplication, unilateral renal agenesis, hyperechogenic kidney, polycystic kidney (including enlarged kidney), ectopic kidney (including pelvic kidney and crossed fused renal ectopia), and horseshoe kidney. These renal abnormalities were defined according to the ISUOG Practice Guidelines. The study was approved by the institutional ethics committee, all parents of the fetuses consented to participate in this study, providing signed informed consent.

Pregnant women underwent amniocentesis between 17 and 34 weeks of pregnancy, and all samples underwent CMA/CNV-seq. Of the 298 CMA/CNV-seq negative participants, only 76 cases agreed to undergo WES testing. Table S1 provides detailed clinical information, including maternal age, gestational week, and diagnostic strategies.

Genomic DNA was extracted from amniotic fluid and peripheral blood samples of biological parents using the Qiagen DNA Blood Midi/Mini Kit (Qiagen GmbH, Hilden, Germany) following the protocol of the manufacturer.

### Genetic testing and data analysis

All samples underwent short tandem repeat (STR) analysis to eliminate maternal contamination for parentage analysis. The DNA was digested, ligated with connectors, amplified, purified, and labeled with biotin. Subsequently, DNA was hybridized to Affymetrix CytoScan 750 K arrays (Affymetrix, Santa Clara, CA, USA) containing a-CGH and SNP probes. Data were analyzed using Chromosome Analysis Software (ChAS, version 4.2.0.80). Qualified libraries for CNV-seq underwent pooling. Subsequently, they were subjected to massively parallel sequencing on the NextSeq 500CN (Illumina, San Diego, USA). WES samples underwent massively parallel sequencing on NanoWES (Berry Genomics, China) and the Novaseq6000 platform (Illumina, San Diego, USA). Raw image files underwent base calling using CASAVA v1.82 to generate raw data. Detected variants were annotated using ANNOVAR (http://annovar.openbioinformatics.org/en/latest/) and the Enliven^®^ Variants Annotation Interpretation System.

Identified variants underwent further analysis using public databases, such as the Database of Genomic Variants (DGV, http://projects.tcag.ca/variation), gnomAD (http://gnomad.broadinstitute.org/), the 1000 Genome Project (http://browser.1000genomes.org), DECIPHER (http://decipher.sanger.ac.uk/), ClinVar (https://www.ncbi.nlm.nih.gov/clinvar/), OMIM (https://www.omim.org), and HPO (https://hpo.jax.org/app/*).* Pathogenicity of genetic variants was assessed according to the American College of Medical Genetics and Genomics (ACMG) guidelines [16, 17]. Statistical comparisons were conducted using the chi-squared test, with a significance level set at *p* < 0.05.

### Optical genome mapping

Optical genome mapping (OGM) was used to detect unbalanced structural variations such as CNVs and balanced chromosomal rearrangements, including translocations and inversions. Additionally, it was capable of fine-tuning the location of breakpoints and determining the orientation of duplicated or inserted segments. In a specific case, the father had 17q12 duplication, while the CNV-normal mother underwent OGM to determine the arrangement of 17q12. Ultra-high molecular weight (UHMW) genomic DNA from whole blood was extracted using the Bionano Prep SP Blood DNA Isolation Kit (80030, Bionano Genomics, San Diego, CA, USA) for OGM. The DNA was labeled with green fluorescence, while the DNA backbone was stained using the Bionano Prep Direct Label and Stain (DLS) Kit (#30206, Bionano Genomics). This labeled DNA was loaded onto the Saphyr chip (Bionano Genomics) for linearization and imaging on the Saphyr instrument, generating over 500 Gb of data per sample. The data underwent analysis using Bionano Solve software (version 3.7) and visualized in Bionano Access v.1.7. This procedure included assembling, aligning to the genome reference (GRCh38), and identifying structural variants.

## Results

### Sample characteristics

Eleven types of fetal renal abnormalities were reported by prenatal ultrasound in 329 unrelated fetuses. This cohort comprised 195 male (59.3%) and 134 female (40.7%) fetuses (Table S1). All participants were of Han Chinese ethnicity from central China’s Hubei province. This study retrospectively analyzed genetic testing results from 329 fetuses, including 207 isolated renal anomalies and 122 non-isolated renal anomalies (Table 1). The age range of pregnant women at prenatal diagnosis was 18 to 46 years, while gestational age ranged from 17 to 34 weeks (Table S1). Non-isolated renal anomalies were those combined with soft ultrasound indicators (echogenic fetal bowel, choroid plexus cysts, polyhydramnios/oligohydramnios, dilated lateral ventricles, and nasal bone anomalies) or other systemic anomalies (cardiovascular, skeletal, and neurological). Among the 329 participants, hydronephrosis was the most common renal anomaly (79 cases), followed by multiple renal anomalies (44 cases), polycystic kidneys (40 cases), unilateral renal agenesis (34 cases), and hyperechogenic kidneys (31 cases) (Table 1). Less common renal anomalies included MCDK (11 cases) and horseshoe kidney (10 cases).

**Table 1:** Distribution of the 329 fetuses with renal anomalies and the diagnostic yield from CMA/CNV-seq

Prenatal renal anomalies (HPO term)	Total cases *N*	Cases diagnosed via CMA/CNV-seq	Non-isolated anomalies		Isolated anomalies	
			Total cases *N*	Cases diagnosed via CMA/CNV-seq	Total cases *N*	Cases diagnosed via CMA/CNV-seq
Hydronephrosis HP:0000126	79	3(3.8%)	38	1(2.6%)	41	2(4.9%)
More than one renal anomaly	44	3(6.8%)	9	2(22.2%)	35	1(2.9%)
Polycystic kidneys HP:0000113	40	1(2.5%)	8	1(12.5%)	32	
Unilateral renal agenesis HP:0000104	34		10		24	
Hyperechogenic kidneys HP:0004719	31	11(35.5%)	19	5(26.3%)	12	6(50%)
Renal duplication HP:0000075	28	3(10.7%)	11	2(18.2%)	17	1(5.9%)
Renal dysplasia HP:0000110	22	4(18.2%)	10	3(30%)	12	1(8.3%)
Renal cysts HP:0000107	16	2(12.5%)	5	1(20%)	11	1(9.1%)
Ectopic kidney HP:0000086	14	1(7.1%)	6	1(16.7%)	8	
MCDK HP:0000003	11	2(18.2%)	2		9	2(22.2%)
Horseshoe kidney HP:0000085	10	1(10%)	4	1(25%)	6	
Total	329	31(9.4%)	122	17(13.9%)	207	14(6.8%)

Abbreviations: HPO, Human Phenotype Ontology; HP, the HPO Term Identifier; MCDK, multicystic dysplastic kidney

### CMA/CNV-seq results

Chromosomal abnormalities were detected in 31 of 329 fetuses with renal anomalies tested using by CMA/CNV-seq, yielding an overall detection rate of 9.4% (31/329) (Table 1). The overall detection rate was significantly higher for non-isolated renal anomalies than for isolated renal anomalies (*p* = 0.03, 13.9% vs. 6.8%). The predominant soft ultrasound markers in positive CMA/CNV-seq cases included amniotic fluid abnormalities (4 cases), echogenic fetal bowel (4 cases), and placental thickening (2 cases). All three positive cases with multiple renal abnormalities exhibited bilateral hyperechogenic kidneys, one with renal dysplasia and two with hydronephrosis.

The detection rate of non-isolated renal anomalies was significantly higher than that in isolated renal anomalies for most types, except for MCDK, hydronephrosis, and hyperechogenic kidneys. MCDK was detected at an 18.2% rate, all within isolated renal anomalies (Table 1). Unilateral renal agenesis (34 cases) showed no pathogenic/likely pathogenic CNVs, indicating a weak association with chromosomal abnormalities. The hyperechogenic kidney group had the highest CMA/CNV-seq detection rate, strongly associated with 17q12 deletion (Table 2).

**Table 2:** Chromosomal abnormalities detected using CMA/CNV-seq in fetuses with renal anomalies

Case ID	Renal anomalies (HPO term)	Other ultrasound findings (HPO term)	CMA/CNV-seq	Size	Classify	De novo/Inheritance	Outcome
**Hyperechogenic kidneys**							
A127^*^	Bilateral hyperechogenic kidneys (HP:0004719)	Single umbilical artery (HP:0001195) Thickened placenta	arr[GRCh37] 5p15.33p15.31(113577_6635886)x1 arr[GRCh37] 10q23.31q26.3(91274502_135426386)x3	6.5 Mb 44 Mb	*P* *P*	ND	TOP
A991	Bilateral hyperechogenic kidneys (HP:0004719)		arr[GRCh37] 7q36.3(156135729_157082329)x3	947 kb	*P*	Maternal	TOP
C0919	Bilateral hyperechogenic kidneys (HP:0004719)		seq[hg19]del(10)(q26.13q26.3)chr10: g.126060000_135440000del	9.38 Mb	*P*	ND	TOP
A441^*^	Bilateral hyperechogenic kidneys (HP:0004719)	Polyhydramnios (HP:0001561)	arr[GRCh37] 17q12(34822465_36404104)x1	1.6 Mb	*P*	*De novo*	TOP
A728	Bilateral hyperechogenic kidneys (HP:0004719)		arr[GRCh37] 17q12(34822466_36243365)x1	1.42 Mb	LP	*De novo*	TOP
A655^*^	Bilateral hyperechogenic kidneys (HP:0004719)	Polyhydramnios (HP:0001561)	arr[GRCh37] 17q12(34822466_36378678)x1	1.56 Mb	*P*	*De novo*	TOP
A753	Bilateral hyperechogenic kidneys (HP:0004719)		arr[GRCh37] 17q12(34822466_36307773)x1	1.5 Mb	*P*	ND	TOP
A593	Bilateral hyperechogenic kidneys (HP:0004719)		arr[GRCh37] 17q12(34822466_36243365)x1	1.4 Mb	*P*	Maternal	LB
C0831	Bilateral hyperechogenic kidneys (HP:0004719)		seq[hg19]del(17)(q12)chr17:g.34800000_36260000del	1.46 Mb	*P*	*De novo*	TOP
C0087^*^	Bilateral hyperechogenic kidneys (HP:0004719)	Hydrops fetalis (HP:0001789) Echogenic fetal bowel (HP:0010943) Pleural effusion (HP:0002202)	seq[hg19]dup(21)(p13q22.3)chr21: g.1_48129895dup	42 Mb	*P*	-	TOP
A193^*^	Bilateral hyperechogenic kidneys (HP:0004719)	Cystic hygroma (HP:0000476) Echogenic fetal bowel (HP:0010943) Decreased fetal movement (HP:0001558)	arr[GRCh37] Xp21.1(31691101_31889320)x0	198 kb	*P*	*De novo*	TOP
**Polycystic kidneys**							
C0549^*^	Enlarged polycystic kidneys (HP:0000113)	Abnormal placenta morphology (HP:0100767)	seq[hg19]del(17)(q12)chr17:g.34800000_36260000del	1.46 Mb	*P*	*De novo*	TOP
**Renal dysplasia**							
A228^*^	Small kidneys (HP:0000089)	Talipes equinovarus (HP:0001762) Intrauterine growth retardation (HP:000151)	arr[GRCh37] 4p16.3(68345_2749375)x1	2.68 Mb	*P*	ND	TOP
C1439^*^	Small kidneys (HP:0000089)	Oligohydramnios (HP:0001562)	seq[hg19]del(4)(p16.3)chr4: g.1220000_2760000del	1.54 Mb	*P*	*De novo*	TOP
A139^*^	Small kidneys (HP:0000089)	Abnormality of the abdominal wall (HP:0004298) Abnormal ear morphology (HP:0031703)	arr[GRCh37] 11q23.3q25(116683755_134937416)x3 arr[GRCh37] 22q11.1q11.21(16888900_20312661)x3	18.3 Mb 3.4 Mb	*P* LP	*De novo* *De novo*	TOP
C0832	Renal dysplasia (HP:0000110)		seq[hg19]del(16)(p11.2)chr16: g.29680000_30200000del	520 kb	LP	Paternal	LB
**Hydronephrosis**							
A140	Abnormal renal collecting system morphology (HP:0004742)		arr[GRCh37] 18p11.32p11.22(136227_10687437)x1 arr[GRCh37] Yq11.21q12(14874542_59032809)x0	10.6 Mb 44 Mb	*P* *P*	ND	TOP
C3046^*^	Abnormal renal collecting system morphology (HP:0004742)	Ventricular septal defect (HP:0001629)	seq[hg19]dup(21)(p13q22.3)chr21:g.1_48129895dup	48 Mb	*P*	-	TOP
A508	Abnormal renal collecting system morphology (HP:0004742)		arr[GRCh37] 22q11.21(18970561_20312661)x3	1.4 Mb	LP	Maternal	LB
**Renal duplication**							
A014^*^	Renal duplication (HP:0000075)	Choroid plexus cyst (HP:0002190)	arr[GRCh37] 8p23.3p23.1(158048_8656658)x1 arr[GRCh37] 9p24.3p21.2(208454_26991513)x3	8.5 Mb 26.8 Mb	*P* *P*	*De novo* Maternal	TOP
A451^*^	Renal duplication (HP:0000075)	Single umbilical artery (HP:0001195)	arr(16)x2 ~ 3	90 Mb	*P*	-	TOP
A232	Renal duplication (HP:0000075)		arr[GRCh37] 17q12(34822466_36243365)x3	1.4 Mb	LP	ND	TOP
**Renal cysts**							
A967^*^	Renal cysts (HP:0000107)	Abnormality of the liver (HP:0001392)	arr[GRCh37] 7q35q36.3(146530551_159119707)x1	12.6 Mb	*P*	*De novo*	TOP
A066	Renal cysts (HP:0000107)		arr(X)x1, (Y)x2	59 Mb	*P*	-	LB
**Multicystic dysplastic kidney**							
C1349	Multicystic kidney dysplasia (HP:0000003)		seq[hg19]del(17)(q12)chr17:g.34820000_36260000del	1.44 Mb	LP	Paternal	TOP
A771	Multicystic kidney dysplasia (HP:0000003)		arr[GRCh37] 22q11.21(18648856_21800471)x1	3.15 Mb	*P*	*De novo*	TOP
**Horseshoe kidney**							
C0476^*^	Horseshoe kidney (HP:0000085)	Ventricular septal defect (HP:0001629) Umbilical vein varix (HP:0030656)	seq[hg19]dup(11)(q23.3q25)chr11: g.116700000_134960000dup seq[hg19]dup(22)(q11.1q11.21)chr22: g.16840000_20260000dup	18.26 Mb 3.42 Mb	*P* LP	ND	TOP
**Ectopic kidney**							
C0475^*^	Crossed fused renal ectopia (HP:0004736)	Oligohydramnios (HP:0001562) Patent foramen ovale (HP:0001655)	seq[hg19]del(1)(q23.3)chr1: g.163620000_164760000del	1.14 Mb	*P*	ND	TOP
**More than one renal anomaly**							
A184^*^	Bilateral hyperechogenic kidneys (HP:0004719) Abnormal renal collecting system morphology (HP:0004742) Fetal pyelectasis (HP:0010945) Hydroureter (HP:0000072)	Echogenic fetal bowel (HP:0010943)	arr[GRCh37] 9q21.33q34.13(89573083_135830937)x3	46.3 Mb	*P*	*De novo*	TOP
A147^*^	Bilateral hyperechogenic kidneys (HP:0004719) Bilateral fetal pyelectasis (HP:0011129)	Echogenic fetal bowel (HP:0010943)	arr[GRCh37] 11q23.3q25(119502793_131881347)x1	12.4 Mb	*P*	*De novo*	TOP
A210	Bilateral hyperechogenic kidneys (HP:0004719) Small kidneys (HP:0000089)		arr[GRCh37] 17q12(34822466_36397279)x1	1.58 Mb	*P*	*De novo*	TOP

Abbreviations: HPO, Human Phenotype Ontology; HP, the HPO Term Identifier. LP, likely pathogenic; P, pathogenic; TOP, termination of pregnancy; ND, not detected; LB, live birth. Asterisk indicate cases of non-isolated renal anomaly

Among the 31 positive cases detected by CMA/CNV-seq, 31 pathogenic/likely pathogenic CNVs, 4 common aneuploidies, and 1 case of trisomy 16 mosaicism (case ID A451) were identified (Table 2). Additionally, 9 cases of 17q12 deletion (29%, 9/31) and 1 case of 17q12 duplication were detected among the 329 fetuses with renal abnormalities in this study. The 31 CNVs contained known hotspot CNVs associated with renal abnormalities, such as three 22q11.2 (one deletion, two duplications), two 4p16.3 (two deletions), and one 16p11.2 deletion. Among these, 14 CNVs were *de novo*, while 7 were inherited from parents in 21 families that underwent parent-of-origin testing. The clinical follow-up of the 31 positive cases showed that 14 pregnancies with *de novo* CNVs were terminated. Among these, 11 were large fragment deletions, while 3 were large fragment duplications. In contrast, pregnancies with inherited CNVs were mostly continued, and carrier parents showed no evidence of disease. Additionally, seven families chose to terminate their pregnancies without determining the CNV origin. Four of the 31 in total were live births. Of the four live births followed up, one (case ID A593) passed away due to brain tumor growth at approximately 1 year of age. The remaining three were followed up until June 28 (8 months to 3 years) without any abnormalities observed.

### WES results

Out of 298 fetuses with non-diagnostic, negative CMA/CNV-seq results, 222 parents declined WES, while WES analysis was performed in 76 cases. Paternity was clearly established in the families receiving trio-WES. The overall WES detection rate was 15.8% (12/76) (Table 3). Among the 12 positive WES cases, seven had renal anomalies combined with systemic anomalies, mainly cardiac, skeletal, and neurological (Table 4). Five WES-positive cases were identified in the isolated group, some presenting with multiple renal findings. In contrast, the 28 non-isolated cases exhibited a significantly higher WES detection rate of 25%, compare to the 10.4% in the 48 cases of the isolated group (25% vs. 10.4%) (Table 3). The WES yield for various renal anomalies differed: hyperechogenic kidney showed a 28.6% yield (2/7), MCDK yielded 20% (1/5), polycystic kidney yielded 18.8% (3/16), unilateral renal agenesis yielded 16.7% (2/12), and multiple renal abnormalities in four cases (22.2%, 4/18). The four cases with multiple renal anomalies identified by WES included two cases of hyperechogenic kidney and enlarged kidney, one case of renal dysplasia with a pelvic kidney, and one case of polycystic kidney with contralateral hydronephrosis.

**Table 3:** WES diagnostic yield in fetal renal abnormalities with negative CMA/CNV-seq results

Prenatal renal anomalies (HPO term)	Total cases *N*	Cases diagnosed by WES	Non-isolated cases		Isolated cases	
			Total cases	Diagnosed cases	Total cases	Diagnosed cases
More than one renal anomaly	18	4(22.2%)	3	1(33.3%)	15	3(20%)
Polycystic kidneys HP:0000113	16	3(18.8%)	3	2(66.7%)	13	1(7.7%)
Unilateral renal agenesis HP:0000104	12	2(16.7%)	3	1(33.3%)	9	1(11.1%)
Hyperechogenic kidneys HP:0004719	7	2(28.6%)	6	2(33.3%)	1	
Hydronephrosis HP:0000126	6		5		1	
MCDK HP:0000003	5	1(20%)	1	1	4	
Ectopic kidney HP:0000086	5		2		3	
Renal duplication HP:0000075	2		2			
Renal dysplasia HP:0000110	2		1		1	
Renal cysts HP:0000107	2		2			
Horseshoe kidney HP:0000085	1				1	
Total	76	12(15.8%)	28	7(25%)	48	5(10.4%)

Abbreviations: HPO, Human Phenotype Ontology; HP, the HPO Term Identifier. MCDK, multicystic dysplastic kidney

**Table 4:** Single nucleotide variants and copy number variants detected via WES in fetuses with renal anomalies

Case ID	Sonographic findings (HPO term)	Gene	Variant	Allele frequency (gnomAD)	Inheritance	Zygosity	Clinical significance	Disease (#OMIM)
5570	Bilateral hyperechogenic kidneys (HP:0004719) Hydrocephalus (HP:0000238) Oligohydramnios (HP:0001562) Abnormality of the bladder (HP:0000014)	*RNU4ATAC*	NR_023343.1: n.51G > A NR_023343.1: n.46G > A	0.000770119 0.000246407	AR	compound het	*P* *P*	Microcephalic osteodysplastic primordial dwarfism, type I OMIM:210,710
4735	Polycystic kidney dysplasia (HP:0000113) Postaxial foot polydactyly (HP:0001830) Ventricular septal defect (HP:0001629)	*TMEM67*	NM_153704.6: c.1353del^a^ p.(E452Kfs*4) NM_153704.6: c.1321 C > T p.(R441C)	0.000163185 5.44484e-05	AR	compound het	LP VUS	Meckel syndrome 3 OMIM:607,361
1774	Multicystic kidney dysplasia (HP:0000003) Pericardial effusion (HP:0001698) Oligohydramnios (HP:0001562)	*NPHP3*	NM_153240.5: c.748 C > T p.(Q250*) NM_153240.5: c.2917 C > T p.(R973*)	0.000 0.000108743	AR	compound het	*P* *P*	Meckel syndrome 7 OMIM:267,010
8250	Unilateral renal agenesis (HP:0000122) Congenital heart defect (HP:0001627) Ventricular septal defect (HP:0001629) Right aortic arch with mirror image branching (HP:0002627)	*CHD7*	NM_017780.4: c.1931dup^a^ p.(K645Efs*31)	0.000	AD	*de novo* het	*P*	CHARGE syndrome OMIM:214,800
2001	Polycystic kidney dysplasia (HP:0000113) Cerebellar vermis hypoplasia (HP:0001320) Polydactyly (HP:0010442)	*CEP290*	NM_025114.4: c.5788 A > T p.(K1930*) NM_025114.4: c.1666dup p.(I556Nfs*20)	0.000 0.00279767	AR	compound het	*P* *P*	Joubert syndrome 5 OMIM:610,188
3008	Unilateral renal agenesis (HP:0000122) Fetal ascites (HP:0001791)	*KMT2D*	NM_003482.4: c.4395dup p.K1466Qfs*25	0.000	AD	*de novo* het	*P*	Kabuki syndrome 1 OMIM:147,920
1199	Enlarged kidney (HP:0000105) Bilateral hyperechogenic kidneys(HP:0004719) Polydactyly (HP:0010442) Intrauterine growth retardation (HP:0001511)	*BBS2*	NM_031885.4: c.700 C > T p.(R234*) NM_031885.4: c.650T > G^a^ p.(F217C)	5.78369e-05 0.000	AR	compound het	LP LP	Bardet-Biedl syndrome 2 OMIM:615,981
7460*	Enlarged kidney (HP:0000105) Bilateral hyperechogenic kidneys (HP:0004719)	*TTC8*	NM_198309.3: c.1222 C > T^a^ p.(Q408*) NM_198309.3: c.529 C > T^a^ p.(Q177*)	0.000 8.83642e-06	AR	compound het	*P* *P*	Bardet-Biedl syndrome 8 OMIM:615,985
2003	Polycystic kidney dysplasia (HP:0000113) Hydronephrosis (HP:0000126) Abnormal renal collecting system morphology (HP:0004742)	*PKD1*	NM_001009944.3: c.6571 C > T p.(R2191C)	0.000192308	AD	ND	LP	Polycystic kidney disease 1 OMIM:173,900
7402	Bilateral hyperechogenic kidneys (HP:0004719) Short femur (HP:0003097) Short humerus (HP:0005792)	*PKD1*	NM_001009944.3: c.3067 C > T p.(Q1023*)	0.000	AD	het (affected mother)	*P*	Polycystic kidney disease 1 OMIM:173,900
1793*	Polycystic kidneys (HP:0000113)	*PKD1*; *RAB26*; *TRAF7*; *CASKIN1*	wes[hg19]chr16p13.3 (2112339–2190358)x1	-	-	-	LP	Polycystic kidney disease 1 OMIM:173,900
**0535**	Pelvic kidney (HP:0000125) Renal dysplasia (HP:0000110)	16p12.2 recurrent region	wes[hg19]chr16p12.2 (21898945–22460969)x1	-	-	-	LP	Recurrent 16p12.1 microdeletion (neurodevelopmental susceptibility locus)

Abbreviations: HPO, Human Phenotype Ontology; HP, the HPO Term Identifier. AD, autosomal dominant; AR, autosomal recessive; Het, heterozygous; Hom, homozygous; LP, likely pathogenic; P, pathogenic. IDs in bold represent cases where CNV was detected. Asterisks indicate cases of isolated renal anomaly

Among the 76 cases, 10 harbored 16 distinct pathogenic variants across nine genes, corresponding to nine OMIM entries for various malformations. These variants comprised seven nonsense variants, four frameshift variants, three missense variants, and two regulatory variants (Table 4). Six cases had compound heterozygous variants, and two were diagnosed using WES-based CNV analysis. Fetal renal abnormalities were associated with pathogenic variants in five ciliary genes (*TMEM67*, *NPHP3*, *CEP290*, *BBS2*, and *TTC8*). Out of the 10 cases, four were inherited as autosomal dominant due to *de novo* variants, while six were autosomal recessive with compound heterozygous variants. Five novel pathogenic/likely pathogenic variants were discovered (Table 4).

### Case study (C0549) reflecting the origin of recurrent microdeletions

**Fig. 1:**
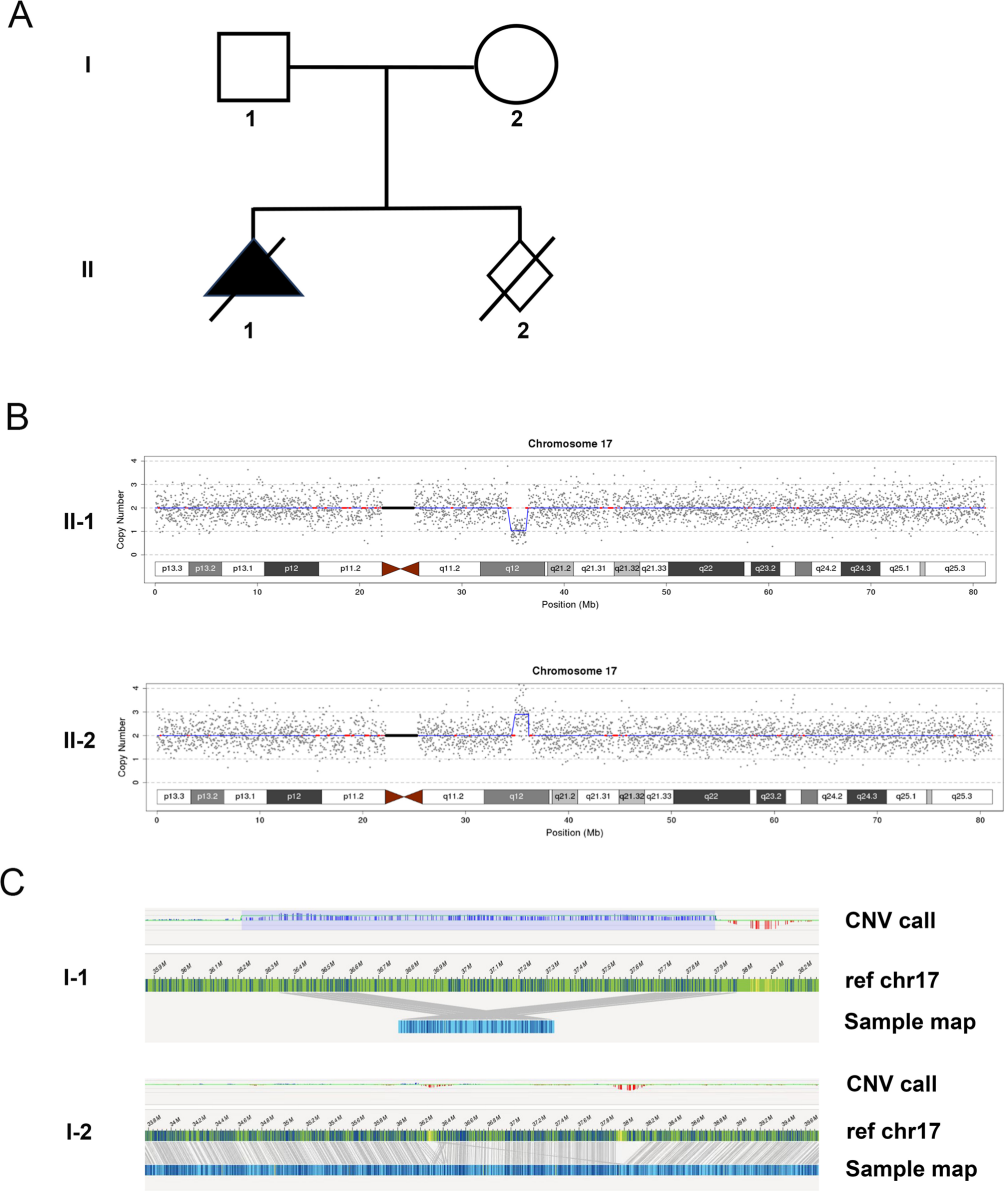
Genetic diagnosis of the family with chromosome 17 abnormalities. (**A**) Pedigree showing renal anomalies in the family. (**B**) Chromosome 17 abnormalities detected in the two fetuses using CNV-seq. (**C**) Detailed diagram of chromosome 17 for the fetal father (I-1) and mother (I-2), respectively

A 33-year-old pregnant woman presented to the Medical Genetics Centre of Hubei Provincial Maternal and Child Health Hospital at 24 + 2 weeks of gestation. Her ultrasound showed fetal right renal enlargement with polycystic changes and mild placental thickening. The nuchal translucency sonogram and cell-free DNA fetal aneuploidy screening were negative. At 25 weeks, amniocentesis was performed for CNV-seq and trio WES on amniotic fluid DNA. The CNV-seq results showed approximately 1.46 Mb fragment deletion in the chr17q12 region, including the 17q12 recurrent autosomal dominant (RCAD) syndrome region with *HNF1B* (Fig. 1A). Trio-WES indicated 17q12 fragment deletion in the fetus, while the father had a duplicated 17q12 fragment. The father showed no renal ultrasound abnormalities. The pregnancy was terminated, and the father did not undergo further testing to clarify the location of the 17q12 repeat insertion.

One year later, the couple conceived spontaneously and chose amniocentesis at 18 weeks. Pre-procedure ultrasound revealed intrauterine fetal death with fetal size equivalent to 14 + 5 weeks. The parents consented to CNV-seq on the umbilical cord, revealing the 17q12 duplication in the second fetus (Fig. 1B).

To understand the outcomes of their two pregnancies, parental blood samples underwent OGM testing. The results showed a “1 + 2” arrangement of 17q12 duplication in the father (one chromosome carrying one 17q12 fragment and the other carrying two 17q12 fragments) (Fig. 1C). Conversely, the mother showed no abnormal changes in chromosome structure or copy number throughout the genome (Fig. 1C). Re-analyzing the trio-WES data revealed that the fetus inherited its single copy of 17q12 from the mother, suggesting the 17q12 deletion originated from the paternal chromosome. Therefore, parental genetic tests imply paternal transmission of the 17q12 duplication in one fetus and a potential predisposition to non-allelic homologous recombination (NAHR) event, resulting in a *de novo* 17q12 microdeletion in the sibling fetus. These genetic findings will inform the family-building choices and future pregnancy management decisions of the couple.

## Discussion

Retrospectively, the CMA/CNV-seq results of 329 fetuses with renal anomalies were analyzed in this study. Chromosomal abnormalities were detected in 31 cases (9.4%). Among 76 CMA/CNV-seq negative fetuses whose parents opted for further molecular testing, monogenic variants were detected via WES in 10 cases (13.2%), while 2 cases (2.6%) were identified through WES-CNV. Additionally, different detection rates were observed for different types of renal abnormalities using CMA/CNV-seq versus WES. Pathogenic CNVs were likely to be identified in cases of renal dysplasia, renal cysts, hydronephrosis, ectopic kidney, and renal duplication. However, no single gene variants were found in these categories. Conversely, only single gene mutations were identified in unilateral renal agenesis. Hyperechogenic kidneys exhibited the highest detection rates for CMA/CNV-seq and WES.

Several recent studies have shown that CMA achieves diagnostic rates of 3.6-13.52% in fetuses with renal anomalies [5–8]. In this retrospective study, we found a diagnostic yield of 9.4% (31/329) by CMA/CNV-seq, which is consistent with the rates reported in these studies. Nevertheless, the utility of WES for the molecular diagnosis of fetuses with renal anomalies remains uncertain. In the PAGE study, a total of 16 fetuses with renal anomalies were detected by WES, and the diagnostic yield was 0 [18]. However, in the Columbia University study, WES was used in 25 fetuses with renal anomalies and the diagnostic yield was 16% [19]. In a Chinese cohort, WES detected monogenic variants in 31 of 160 fetuses (19.38%) with non-diagnostic CMA results [6]. In addition, a molecular diagnosis rate of 12.3% (20/163) was achieved in the 163 fetus-parental trios with normal findings upon karyotyping and CMA [12]. Here we used WES in CMA/CNV-seq negative fetuses and the overall proportion of diagnostic genetic variants was 15.8% (12/76), which is similar to the Columbia University study. The different diagnostic rates could be affected by many factors, such as cohort size, variant interpretation criteria, diversity of renal anomalies and inclusion criteria.

In close association with hyperechogenic kidneys, 17q12 deletion was observed, which is consistent with previous study findings [20–23]. Among the cohort, 9 cases of 17q12 deletion were detected, with seven of them (77.8%, 7/9) exhibiting bilateral hyperechogenic kidneys (with two cases of combined polyhydramnios). Additionally, 17q12 deletion was found in two more fetuses, one presenting with right polycystic kidneys and mild placental thickening and the other with MCDK. These findings highlight the clinical heterogeneity in the prenatal presentation of fetuses with 17q12 deletion. The 17q12 deletion is the primary cause of prenatal renal anomalies in fetuses, mostly occurring *de novo* [24, 25]. Patients with 17q12 microdeletion syndrome exhibit a variable clinical phenotype characterized by kidney and urinary tract structural or functional abnormalities [26–28]. Understanding the origin of 17q12 microdeletion is important for prognosis and pregnancy decision-making.

The association between isolated renal agenesis and CNV remains uncertain. Hu et al. reported no chromosomal abnormalities in 35 fetuses of isolated renal agenesis (0/35) [8]. Lena et al. identified only two pathogenic CNVs in 81 fetuses of isolated renal agenesis [29]. However, Su et al. identified a relatively higher rate of pathogenic CNVs (11.53%, 9/78) in fetuses with isolated renal agenesis [7]. This study, involving 34 fetuses with isolated unilateral renal agenesis, found no CNVs, supporting the conclusion that CNVs are not associated with isolated renal agenesis.

Two CNVs were identified using the CNV calling algorithm in WES data analysis. This suggests that the WES-based CNV calling tool could be used to effectively enhance diagnostic accuracy and strengthen WES as a prenatal diagnostic method.

Oligohydramnios is a recognized risk factor associated with adverse neonatal, fetal, and maternal outcomes, including the need for surgery, impaired renal function, and hemorrhage [30–32]. Liu et al. found single gene variants in five cases exhibiting renal anomalies and oligohydramnios, but found no chromosomal abnormalities [6]. However, this study revealed chromosomal abnormalities using CNV-seq in two of six fetuses with renal anomalies and oligohydramnios. Additionally, single gene variants were found using WES in two of the remaining four cases. These results suggest that combing CMA/CNV-seq with WES is advisable for fetal renal anomalies associated with oligohydramnios.

Beyond genetic associations, our study highlights critical clinical indicators and risk factors for fetal renal anomalies. Ultrasonographic markers such as hyperechogenic kidneys (with 35.5% CMA/CNV-seq yield in our cohort), oligohydramnios (with pathogenic variants identified in 4 out of 6 cases), and extra-renal anomalies (e.g., cardiac defects) should raise suspicion for underlying genetic disorders. Additionally, maternal factors, including pre-gestational diabetes and hypertension [33–35], as well as family history of renal malformations [36], significantly elevate the risk. Integrating these features into testing protocols-prioritizing WES for cases with oligohydramnios or syndromic features-optimizes prenatal diagnosis, as demonstrated in our cohort.

## Conclusion

In this study, we retrospectively analyzed the prenatal diagnostic results of 329 fetuses with renal anomalies and found that CMA/CNV-seq had a detection rate of 9.4%, while WES revealed further abnormalities with a detection rate of 15.8%. Frequent mutations in genes encoding ciliary proteins were found in these fetuses. Depending on the case, CMA/CNV-seq or WES testing may be preferred to identify the genetic cause. As knowledge advances, this study and similar ones will contribute to defining the optimal genetic testing strategy for specific types of renal anomalies. These findings have implications for diagnosing kidney abnormalities in fetuses and developing practical guidelines.

## Supplementary Information

Below is the link to the electronic supplementary material.

Supplementary Material 1

## Data Availability

The details of the variant analyzed during the current study are deposited in the ClinVar repository, which is searchable by searching for an SCV accession provided in the summary report. A summary report of our successfully processed data: https://submit.ncbi.nlm.nih.gov/api/2.0/files/t2z0q3g8/sub14386501__100__submitter_report_b.txt/?format=attachment. The raw data for the participants are not publicly available due to privacy or ethical restrictions. These data are available from the corresponding author on reasonable request.

## Abbreviations

WES: Whole exome sequencing
CMA: Chromosomal microarray analysis
OGM: Optical genome mapping
HPO: Human phenotype ontology
CNVs: Copy number variants
ACMG: American college of medical genetics and genomics
VUS: Variants of uncertain significance
LP: Likely pathogenic
P: Pathogenic
AD: Autosomal dominant
AR: Autosomal recessive
Het: Heterozygous
Hom: Homozygous
