# Supplementary material for: Prenatal diagnosis of fetuses with renal abnormalities: a retrospective analysis of 329 Chinese cases
Source: Orphanet J Rare Dis. 2025 Sep 24;20:486. doi: 10.1186/s13023-025-04001-x (PMC12462046; doi:10.1186/s13023-025-04001-x)
Supplement: Supplementary file 1 — Supplementary Material 1 [file 13023_2025_4001_MOESM1_ESM.docx]

**Table S1** Clinical details of 329 fetuses with renal anomalies

| **Renal anomaly** | **Case ID** | **Gender** | **MA(*y*)** | **GA at AMN(*w*)** | **Ultrasound findings** | **Prenatal testing** |
| --- | --- | --- | --- | --- | --- | --- |
| Polycystic kidneys | A470 | M | 31 | 23 | Left polycystic kidney | Karyotype/CMA/WES |
|  | A074 | M | 26 | 23 | Right polycystic kidney | Karyotype/CMA/WES |
|  | A993 | F | 26 | 18 | Left polycystic kidney | Karyotype/CMA/WES |
|  | 4713 | F | 27 | 19 | Left polycystic kidney | Karyotype/CNV-seq/WES |
|  | 1793 | M | 26 | 27 | Polycystic kidneys | Karyotype/CNV-seq/WES |
|  | 0865 | M | 31 | 23 | Left polycystic kidney | Karyotype/CNV-seq/WES |
|  | 0964 | M | 28 | 23 | Right polycystic kidney | Karyotype/CNV-seq/WES |
|  | 1466 | M | 34 | 22 | Right polycystic kidney, left choroid plexus cyst, polyhydramnios | Karyotype/CNV-seq/WES |
|  | 1592 | F | 28 | 24 | Left polycystic kidney | Karyotype/CNV-seq/WES |
|  | 2128 | M | 33 | 22 | Polycystic kidneys | Karyotype/CNV-seq/WES |
|  | 2160 | F | 36 | 26 | Right polycystic kidney | Karyotype/CNV-seq/WES |
|  | 2285 | F | 35 | 22 | Right polycystic kidney | Karyotype/CNV-seq/WES |
|  | 4735 | M | 29 | 24 | Polycystic kidneys, postaxial foot polydactyly, ventricular septal defect | Karyotype/CNV-seq/WES |
|  | 2001 | M | 26 | 22 | Polycystic kidneys, cerebellar vermis hypoplasia, polydactyly | Karyotype/CNV-seq/WES |
|  | 0028 | F | 26 | 22 | Left polycystic kidney | Karyotype/CNV-seq/WES |
|  | 0237 | M | 32 | 24 | Right polycystic kidney | Karyotype/CNV-seq/WES |
|  | A095 | F | 28 | 25 | Left polycystic kidney, polyhydramnios | Karyotype/CMA |
|  | A741 | M | 32 | 23 | Right polycystic kidney | Karyotype/CMA |
|  | A826 | M | 28 | 23 | Polycystic kidneys | Karyotype/CMA |
|  | A042 | F | 35 | 30 | Right polycystic kidney | CMA |
|  | A269 | F | 26 | 25 | Right polycystic kidney | Karyotype/CMA |
|  | A389 | M | 28 | 25 | Right polycystic kidney | Karyotype/CMA |
|  | A573 | M | 23 | 23 | Right polycystic kidney | Karyotype/CMA |
|  | A638 | F | 33 | 24 | Left polycystic kidney | Karyotype/CMA |
|  | A796 | F | 32 | 27 | Right polycystic kidney | Karyotype/CMA |
|  | A948 | M | 29 | 25 | Left polycystic kidney, abnormal nasal skeleton morphology, polyhydramnios | Karyotype/CMA |
|  | A170 | F | 29 | 28 | Polycystic kidneys | Karyotype/CMA |
|  | A451 | M | 29 | 22 | Right polycystic kidney | Karyotype/CMA |
|  | A510 | F | 28 | 24 | Left polycystic kidney | Karyotype/CMA |
|  | A567 | M | 30 | 25 | Polycystic kidneys | Karyotype/CMA |
|  | A006 | M | 29 | 31 | Left polycystic kidney | CMA |
|  | A034 | F | 28 | 19 | Polycystic kidneys | Karyotype/CMA |
|  | A162 | F | 25 | 24 | Left polycystic kidney, hypoplasia involving the nose | Karyotype/CMA |
|  | A393 | M | 25 | 23 | Polycystic kidneys, choroid plexus cysts, polyhydramnios | Karyotype/CMA |
|  | A220 | M | 26 | 22 | Left polycystic kidney | Karyotype/CMA |
|  | A479 | F | 29 | 24 | Left polycystic kidney | Karyotype/CMA |
|  | A875 | M | 34 | 25 | Right polycystic kidney | Karyotype/CMA |
|  | 1724 | M | 26 | 29 | Polycystic kidneys | CMA |
|  | C0549 | F | 33 | 25 | Right polycystic kidney, abnormal placenta morphology | Karyotype/CNV-seq/WES |
|  | 0386 | F | 36 | 30 | Polycystic kidneys | CMA |
| Unilateral renal agenesis | A701 | M | 25 | 25 | Left renal agenesis, congenital heart defect, ventricular septal defect, right aortic arch with mirror image branching | Karyotype/CMA/WES |
|  | A645 | M | 32 | 30 | Left renal agenesis | CMA/WES |
|  | 6324 | F | 31 | 20 | Left renal agenesis | Karyotype/CMA/WES |
|  | 0197 | M | 33 | 22 | Right renal agenesis, left heart strong spot | Karyotype/CMA/WES |
|  | 0321 | F | 33 | 24 | Right renal agenesis | Karyotype/CMA/WES |
|  | 1901 | F | 29 | 24 | Right renal agenesis | Karyotype/CNV-seq/WES |
|  | 2046 | M | 27 | 23 | Left renal agenesis | Karyotype/CNV-seq/WES |
|  | 2079 | M | 25 | 26 | Left renal agenesis | Karyotype/CNV-seq/WES |
|  | 2229 | F | 27 | 27 | Left renal agenesis | Karyotype/CNV-seq/WES |
|  | 0029 | F | 28 | 25 | Right renal agenesis | Karyotype/CNV-seq/WES |
|  | 3008 | M | 32 | 23 | Right renal agenesis | Karyotype/CNV-seq/WES |
|  | 0500 | M | 32 | 23 | Left renal agenesis, ventricular septal defect | Karyotype/CNV-seq/WES |
|  | A577 | F | 31 | 24 | Right renal agenesis | Karyotype/CMA |
|  | A396 | F | 28 | 30 | Left renal agenesis | CMA |
|  | A683 | M | 28 | 30 | Right renal agenesis, single umbilical artery | CMA |
|  | A804 | M | 28 | 22 | Right renal agenesis | Karyotype/CMA |
|  | A446 | M | 31 | 24 | Left renal agenesis, single umbilical artery | Karyotype/CMA |
|  | A565 | F | 25 | 25 | Right renal agenesis, right subclavian artery vagus | Karyotype/CMA |
|  | A632 | F | 25 | 23 | Right renal agenesis, strong light spot in the left ventricle | Karyotype/CMA |
|  | A771 | M | 24 | 23 | Right renal agenesis, single umbilical artery | Karyotype/CMA |
|  | A058 | M | 30 | 24 | Left renal agenesis, strong light spot | Karyotype/CMA |
|  | A174 | F | 27 | 24 | Left renal agenesis | Karyotype/CMA |
|  | A246 | F | 23 | 24 | Left renal agenesis | Karyotype/CMA |
|  | A626 | F | 34 | 20 | Right renal agenesis | Karyotype/CMA |
|  | A925 | M | 32 | 24 | Right renal agenesis, choroid plexus cysts | Karyotype/CMA |
|  | A037 | M | 32 | 23 | Left renal agenesis | Karyotype/CMA |
|  | A301 | F | 28 | 26 | Right renal agenesis | Karyotype/CMA |
|  | A489 | F | 30 | 23 | Left renal agenesis | Karyotype/CMA |
|  | A546 | F | 28 | 23 | Left renal agenesis | Karyotype/CMA |
|  | 0119 | M | 39 | 29 | Left renal agenesis | CMA |
|  | 0274 | F | 40 | 28 | Right renal agenesis | CMA |
|  | 1188 | M | 29 | 24 | Right renal agenesis | Karyotype/CMA |
|  | 1788 | M | 32 | 22 | Right renal agenesis | Karyotype/CNV-seq |
|  | 2560 | M | 27 | 24 | Left renal agenesis | Karyotype/CNV-seq |
| Hyperechogenic kidneys | 0130 | F | 32 | 17 | Bilateral hyperechogenic kidneys, ventricular septal defect, fetal choroid plexus cysts | Karyotype/CMA/WES |
|  | 7402 | M | 32 | 29 | Bilateral hyperechogenic kidneys, short femur, short humerus | CNV-seq/WES |
|  | 1706 | F | 25 | 29 | Bilateral hyperechogenic kidneys | CNV-seq/WES |
|  | 5570 | M | 26 | 29 | Bilateral hyperechogenic kidneys, hydrocephalus, oligohydramnios, abnormality of the bladder | CNV-seq/WES |
|  | 0255 | F | 31 | 28 | Bilateral hyperechogenic kidneys, polyhydramnios | Karyotype/CNV-seq/WES |
|  | 0405 | M | 41 | 24 | Bilateral hyperechogenic kidneys, echogenic fetal bowel | Karyotype/CNV-seq/WES |
|  | 0503 | F | 31 | 24 | Bilateral hyperechogenic kidneys, echogenic fetal bowel | Karyotype/CNV-seq/WES |
|  | A593 | M | 24 | 25 | Bilateral hyperechogenic kidneys | Karyotype/CMA |
|  | A441 | M | 25 | 30 | Bilateral hyperechogenic kidneys, polyhydramnios | CMA |
|  | A189 | F | 36 | 22 | Bilateral hyperechogenic kidneys, echogenic fetal bowel | Karyotype/CMA |
|  | A222 | M | 24 | 24 | Bilateral hyperechogenic kidneys | Karyotype/CMA |
|  | A193 | M | 33 | 18 | Bilateral hyperechogenic kidneys, echogenic fetal bowel | Karyotype/CMA |
|  | A728 | F | 23 | 24 | Bilateral hyperechogenic kidneys | Karyotype/CMA |
|  | A022 | F | 31 | 25 | Bilateral hyperechogenic kidneys, single umbilical artery | Karyotype/CMA |
|  | A655 | F | 26 | 28 | Bilateral hyperechogenic kidneys, polyhydramnios | CMA |
|  | A753 | M | 31 | 24 | Bilateral hyperechogenic kidneys | Karyotype/CMA |
|  | A991 | M | 28 | 23 | Unilateral kidneys cortex hyperechogenic | Karyotype/CMA |
|  | A058 | F | 28 | 23 | Bilateral hyperechogenic kidneys | Karyotype/CMA |
|  | A127 | F | 29 | 27 | Bilateral hyperechogenic kidneys, single umbilical artery | Karyotype/CMA |
|  | A138 | F | 29 | 23 | Bilateral hyperechogenic kidneys, choroid plexus cysts | Karyotype/CMA |
|  | C0919 | F | 35 | 22 | Bilateral hyperechogenic kidneys | Karyotype/CNV-seq |
|  | A138 | F | 46 | 23 | Bilateral hyperechogenic kidneys, echogenic fetal bowel | Karyotype/CMA |
|  | 2262 | M | 24 | 20 | Bilateral hyperechogenic kidneys | Karyotype/CNV-seq |
|  | C0831 | M | 28 | 25 | Bilateral hyperechogenic kidneys | Karyotype/CNV-seq |
|  | C0087 | M | 35 | 18 | Bilateral hyperechogenic kidneys, hydrops fetalis, echogenic fetal bowel; pleural effusion | Karyotype/CNV-seq |
|  | C0088 | M | 35 | 18 | Bilateral hyperechogenic kidneys, echogenic fetal bowel, choroid plexus cysts | Karyotype/CNV-seq |
|  | 0137 | F | 29 | 18 | Bilateral hyperechogenic kidneys | Karyotype/CNV-seq |
|  | 0145 | F | 36 | 23 | Bilateral hyperechogenic kidneys | Karyotype/CNV-seq |
|  | 0221 | F | 31 | 25 | Bilateral hyperechogenic kidneys, echogenic fetal bowel | Karyotype/CNV-seq |
|  | 0411 | M | 32 | 31 | Bilateral hyperechogenic kidneys, echogenic fetal bowel | CMA |
|  | 0645 | M | 35 | 30 | Bilateral hyperechogenic kidneys, echogenic fetal bowel | CNV-seq |
| Hydronephrosis | A957 | M | 29 | 27 | Hydronephrosis, fetal hydrothorax | Karyotype/CMA/WES |
|  | 0668 | F | 26 | 25 | Hydronephrosis, left heart strong spot | Karyotype/CNV-seq/WES |
|  | 0283 | M | 30 | 17 | Ureterectasia | Karyotype/CMA/WES |
|  | 1271 | M | 29 | 28 | Right hydronephrosis, ureterectasia, polyhydramnios | Karyotype/CNV-seq/WES |
|  | 1817 | M | 33 | 28 | Renal collection system separation, thickened placenta | Karyotype/CNV-seq/WES |
|  | 0114 | F | 29 | 24 | Hydronephrosis, fetal hydrothorax, lateral ventricle dilatation | Karyotype/CMA/WES |
|  | A820 | F | 23 | 22 | Pyelectasis | Karyotype/CMA |
|  | A031 | M | 28 | 17 | Hydronephrosis, ureterectasia | Karyotype/CMA |
|  | A140 | M | 27 | 26 | Hydronephrosis | Karyotype/CMA |
|  | A234 | F | 27 | 27 | Hydronephrosis | Karyotype/CMA |
|  | A389 | M | 30 | 27 | Pyelectasis | Karyotype/CMA |
|  | A428 | M | 30 | 34 | Hydronephrosis, choroid plexus cysts | CMA |
|  | A508 | F | 29 | 22 | Hydronephrosis | Karyotype/CMA |
|  | A660 | M | 28 | 24 | Hydronephrosis | Karyotype/CMA |
|  | A706 | M | 35 | 24 | Hydronephrosis, strong light spot in the left ventricle | Karyotype/CMA |
|  | A259 | M | 28 | 25 | Hydronephrosis, pyelectasis | Karyotype/CMA |
|  | A317 | M | 31 | 31 | Hydronephrosis, widen cerebellar bulbar cisterna | CMA |
|  | A527 | F | 39 | 24 | Pyelectasis, choroid plexus cysts | Karyotype/CMA |
|  | A588 | F | 27 | 23 | Hydronephrosis, ureterectasia | Karyotype/CMA |
|  | A736 | M | 28 | 24 | Hydronephrosis | Karyotype/CMA |
|  | A754 | F | 38 | 31 | Hydronephrosis | CMA |
|  | A020 | M | 33 | 25 | Hydronephrosis, ureterectasia | Karyotype/CMA |
|  | A195 | M | 33 | 23 | Hydronephrosis, strong light spot in the left ventricle | Karyotype/CMA |
|  | A369 | M | 28 | 18 | Hydronephrosis | Karyotype/CMA |
|  | A370 | F | 30 | 30 | Hydronephrosis, pyelectasis, lateral ventricle dilatation, polyhydramnios | CMA |
|  | A698 | F | 24 | 26 | Hydronephrosis | Karyotype/CMA |
|  | A730 | M | 38 | 18 | Hydronephrosis | Karyotype/CMA |
|  | A755 | M | 37 | 25 | Pyelectasis, lateral ventricle dilatation | Karyotype/CMA |
|  | A857 | F | 26 | 29 | Hydronephrosis, strong light spot in the left ventricle | CMA |
|  | A913 | M | 31 | 26 | Hydronephrosis | Karyotype/CMA |
|  | A990 | M | 18 | 31 | Hydronephrosis, tricuspid regurgitation | Karyotype/CMA |
|  | A026 | F | 26 | 18 | Hydronephrosis, cystic hygroma, tricuspid regurgitation | Karyotype/CMA |
|  | A027 | M | 25 | 29 | Hydronephrosis, pyelectasis | CMA |
|  | A231 | M | 32 | 28 | Hydronephrosis, ureterectasia | Karyotype/CMA |
|  | A287 | M | 28 | 26 | Hydronephrosis, lateral ventricle dilatation | Karyotype/CMA |
|  | A610 | F | 26 | 30 | Hydronephrosis, ureterectasia | CMA |
|  | A951 | F | 31 | 28 | Hydronephrosis | Karyotype/CMA |
|  | A962 | M | 35 | 24 | Hydronephrosis, lateral ventricle dilatation | Karyotype/CMA |
|  | A097 | M | 37 | 25 | Hydronephrosis, ureterectasia | Karyotype/CMA |
|  | A132 | M | 33 | 22 | Hydronephrosis | Karyotype/CMA |
|  | A403 | F | 28 | 28 | Hydronephrosis | Karyotype/CMA |
|  | A428 | F | 30 | 29 | Hydronephrosis, ventricular septal defect | Karyotype/CMA |
|  | A758 | M | 26 | 29 | Hydronephrosis | CMA |
|  | A772 | F | 31 | 22 | Hydronephrosis, ventricular septal defect | Karyotype/CMA |
|  | A867 | M | 27 | 31 | Hydronephrosis | CMA |
|  | A188 | M | 30 | 23 | Pyelectasis | Karyotype/CMA |
|  | A387 | M | 31 | 18 | Hydronephrosis | Karyotype/CMA |
|  | A405 | M | 27 | 29 | Hydronephrosis | Karyotype/CMA |
|  | A481 | M | 26 | 31 | Hydronephrosis | CMA |
|  | A721 | M | 33 | 30 | Hydronephrosis, lateral ventricle dilatation | CMA |
|  | A799 | M | 26 | 25 | Hydronephrosis | Karyotype/CMA |
|  | A959 | M | 27 | 26 | Hydronephrosis, pyelectasis | Karyotype/CMA |
|  | A143 | F | 30 | 23 | Hydronephrosis, strong light spot in the left ventricle | Karyotype/CMA |
|  | A226 | M | 33 | 24 | Hydronephrosis | Karyotype/CMA |
|  | A648 | M | 33 | 26 | Hydronephrosis | Karyotype/CMA |
|  | C0822 | F | 25 | 28 | Hydronephrosis, genital abnormality | Karyotype/CNV-seq |
|  | C0865 | F | 31 | 22 | Hydronephrosis | Karyotype/CNV-seq |
|  | C1163 | M | 32 | 24 | Hydronephrosis | Karyotype/CNV-seq |
|  | A027 | M | 42 | 29 | Pyelectasis, polyhydramnios | CMA |
|  | A073 | M | 29 | 31 | Hydronephrosis | CMA |
|  | 1767 | F | 31 | 21 | Hydronephrosis, choroid plexus cysts | Karyotype/CMA |
|  | 1949 | F | 33 | 23 | Hydronephrosis, choroid plexus cysts, strong light spot in the left ventricle | Karyotype/CNV-seq |
|  | A183 | M | 28 | 23 | Hydronephrosis, ureterectasia | Karyotype/CMA |
|  | 2057 | M | 39 | 18 | Hydronephrosis | Karyotype/CNV-seq |
|  | A228 | F | 28 | 26 | Pyelectasis, single umbilical artery | Karyotype/CMA |
|  | 2333 | M | 29 | 22 | Hydronephrosis, tricuspid regurgitation, strong light spot in the left ventricle | Karyotype/CNV-seq |
|  | 2418 | F | 29 | 22 | Hydronephrosis, strong light spot in the left ventricle | Karyotype/CNV-seq |
|  | A324 | M | 28 | 31 | Hydronephrosis, echogenic fetal bowel | CMA |
|  | 2547 | F | 38 | 25 | Hydronephrosis, tricuspid regurgitation, ventricular septal defect | Karyotype/CNV-seq |
|  | 2550 | M | 30 | 22 | Hydronephrosis | Karyotype/CNV-seq |
|  | A405 | M | 35 | 30 | Hydronephrosis, ventricular septal defect | CMA |
|  | C0077 | M | 26 | 23 | Pyelectasis, strong light spot in the left ventricle | Karyotype/CNV-seq |
|  | A442 | F | 28 | 31 | Hydronephrosis, choroid plexus cysts | CMA |
|  | C0283 | M | 32 | 32 | Hydronephrosis, polyhydramnios | CNV-seq |
|  | A474 | M | 35 | 29 | Hydronephrosis | CMA |
|  | C0609 | F | 31 | 30 | Pyelectasis, strong light spot in the left ventricle | CNV-seq |
|  | 0678 | F | 29 | 29 | Hydronephrosis, pyelectasis | CNV-seq |
|  | 0679 | F | 33 | 23 | Hydronephrosis, hypoplasia involving the nose | Karyotype/CNV-seq |
|  | 0720 | M | 31 | 23 | Hydronephrosis, pyelectasis | Karyotype/CNV-seq |
| Multicystic dysplastic kidney | 1235 | M | 30 | 29 | Right multicystic dysplastic kidney | CNV-seq/WES |
|  | 1836 | M | 34 | 22 | Left multicystic dysplastic kidney | Karyotype/CNV-seq/WES |
|  | 2378 | F | 37 | 19 | Left multicystic dysplastic kidney | Karyotype/CNV-seq/WES |
|  | 2627 | M | 29 | 24 | Right multicystic dysplastic kidney | Karyotype/CNV-seq/WES |
|  | 1774 | F | 28 | 24 | Right multicystic dysplastic kidney, pericardial effusion, oligohydramnios | Karyotype/CNV-seq/WES |
|  | A330 | M | 25 | 30 | Left multicystic dysplastic kidney, ventricular septal defect | CMA |
|  | A771 | F | 33 | 25 | Left multicystic dysplastic kidney | Karyotype/CMA |
|  | A502 | F | 28 | 21 | Right multicystic dysplastic kidney | Karyotype/CMA |
|  | A672 | M | 36 | 23 | Left multicystic dysplastic kidney | Karyotype/CMA |
|  | C1349 | M | 31 | 25 | Right multicystic dysplastic kidney | Karyotype/CNV-seq |
|  | C0641 | F | 38 | 24 | Left multicystic dysplastic kidney | Karyotype/CNV-seq |
| Ectopic kidney | A969 | M | 27 | 24 | Pelvic kidney | Karyotype/CMA/WES |
|  | A353 | M | 30 | 24 | Right pelvic kidney | Karyotype/CMA/WES |
|  | 2045 | M | 26 | 23 | Left pelvic kidney | Karyotype/CMA/WES |
|  | 2390 | F | 34 | 26 | Left pelvic kidney, single umbilical artery | Karyotype/CNV-seq/WES |
|  | 2613 | M | 33 | 31 | Crossed fused renal ectopia, single umbilical artery, ventricular septal defect, polyhydramnios | CNV-seq/WES |
|  | A448 | M | 35 | 24 | Left pelvic kidney | Karyotype/CMA |
|  | A329 | F | 31 | 31 | Left pelvic kidney, single umbilical artery | CMA |
|  | A340 | M | 36 | 24 | Right pelvic kidney | Karyotype/CMA |
|  | A418 | M | 33 | 24 | Ectopic pelvic fusion kidney | Karyotype/CMA |
|  | A778 | F | 32 | 24 | Right pelvic kidney, polyhydramnios | Karyotype/CMA |
|  | A595 | M | 31 | 22 | Ectopic pelvic kidney | Karyotype/CMA |
|  | C0475 | F | 35 | 25 | Crossed fused renal ectopia, oligohydramnios | Karyotype/CNV-seq |
|  | C0947 | F | 28 | 24 | Right pelvic kidney, echogenic fetal bowel | Karyotype/CNV-seq |
|  | C1065 | M | 33 | 25 | Ectopic kidney | Karyotype/CNV-seq |
| Horseshoe kidney | C2609 | F | 30 | 27 | Horseshoe kidney | Karyotype/CNV-seq/WES |
|  | A235 | M | 28 | 23 | Horseshoe kidney | Karyotype/CMA |
|  | A458 | M | 30 | 29 | Horseshoe kidney | Karyotype/CMA |
|  | A654 | F | 29 | 31 | Horseshoe kidney, intrauterine growth retardation | CMA |
|  | A899 | M | 32 | 22 | Horseshoe kidney, oligohydramnios | Karyotype/CMA |
|  | A940 | M | 23 | 26 | Horseshoe kidney, tricuspid regurgitation | Karyotype/CMA |
|  | A157 | M | 27 | 22 | Horseshoe kidney | Karyotype/CMA |
|  | A269 | F | 27 | 22 | Horseshoe kidney | Karyotype/CMA |
|  | A686 | M | 29 | 23 | Horseshoe kidney | Karyotype/CMA |
|  | C0476 | M | 38 | 23 | Horseshoe kidney, ventricular septal defect | Karyotype/CNV-seq |
| Renal dysplasia | A704 | F | 26 | 29 | Right renal dysplasia | CMA/WES |
|  | 2549 | F | 27 | 25 | Left renal dysplasia, absent fetal nasal bone | Karyotype/CMA/WES |
|  | A817 | M | 28 | 23 | Right renal dysplasia | Karyotype/CMA |
|  | A587 | M | 35 | 19 | Renal dysplasia | Karyotype/CMA |
|  | A617 | M | 27 | 25 | Renal dysplasia, oligohydramnios | Karyotype/CMA |
|  | A732 | M | 33 | 24 | Renal dysplasia | Karyotype/CMA |
|  | A652 | F | 28 | 24 | Right renal dysplasia | Karyotype/CMA |
|  | A183 | M | 30 | 25 | Left renal dysplasia | Karyotype/CMA |
|  | A211 | M | 32 | 23 | Renal dysplasia, increased nuchal translucency | Karyotype/CMA |
|  | A266 | M | 26 | 23 | Renal dysplasia | Karyotype/CMA |
|  | A360 | F | 23 | 23 | Renal dysplasia, absent fetal nasal bone | Karyotype/CMA |
|  | A228 | F | 35 | 25 | Small kidneys, talipes equinovarus, intrauterine growth retardation | Karyotype/CMA |
|  | A578 | M | 31 | 27 | Right renal dysplasia | Karyotype/CMA |
|  | A437 | M | 29 | 24 | Left renal dysplasia | Karyotype/CMA |
|  | A783 | F | 28 | 24 | Right renal dysplasia, dilated cerebral ventricles | Karyotype/CMA |
|  | A139 | F | 31 | 23 | Small kidneys, abnormality of the abdominal wall, abnormal ear morphology | Karyotype/CMA |
|  | C0832 | M | 27 | 27 | Left renal dysplasia | Karyotype/CNV-seq |
|  | C1272 | F | 32 | 24 | Renal dysplasia, abnormal nasal skeleton morphology | Karyotype/CNV-seq |
|  | C1745 | F | 24 | 23 | Left renal dysplasia | Karyotype/CNV-seq |
|  | A182 | F | 31 | 29 | Right renal dysplasia, enlarged liver | Karyotype/CMA |
|  | A283 | M | 32 | 24 | Right renal dysplasia | Karyotype/CMA |
|  | C1439 | M | 28 | 30 | Small kidneys, oligohydramnios | CNV-seq |
| Renal cysts | 0486 | M | 34 | 24 | Left renal cyst, right hand polydactyly, dilated cerebral ventricles | Karyotype/CNV-seq/WES |
|  | 0576 | M | 31 | 18 | Left renal cyst, single umbilical artery, scoliosis | Karyotype/CNV-seq/WES |
|  | A967 | F | 31 | 25 | Left renal cyst, strong light spots in the liver | Karyotype/CMA |
|  | A066 | M | 27 | 25 | Right renal cyst | Karyotype/CMA |
|  | A677 | F | 32 | 23 | Right renal cyst | Karyotype/CMA |
|  | A789 | M | 29 | 29 | Right renal cyst | CMA |
|  | A241 | M | 27 | 24 | Right renal cyst | Karyotype/CMA |
|  | A543 | F | 32 | 27 | Right renal cyst | Karyotype/CMA |
|  | A026 | F | 25 | 24 | Right renal cyst | Karyotype/CMA |
|  | A523 | M | 29 | 29 | Left renal cyst, strong light spot in the left ventricle | CMA |
|  | C1039 | F | 29 | 23 | Left renal cyst | Karyotype/CNV-seq |
|  | A926 | M | 25 | 24 | Right renal cyst | Karyotype/CMA |
|  | C1297 | M | 30 | 23 | Right renal cyst, thickened placenta | Karyotype/CNV-seq |
|  | C1412 | F | 28 | 26 | Left renal cyst | Karyotype/CNV-seq |
|  | C1451 | M | 32 | 22 | Right renal cyst | Karyotype/CNV-seq |
|  | A200 | M | 29 | 18 | Left renal cyst | Karyotype/CMA |
| Renal duplication | A135 | M | 33 | 29 | Left duplicated kidney, polyhydramnios | CMA/WES |
|  | 8247 | M | 30 | 20 | Right duplicated kidney, dilated cerebral ventricles, ventricular septal defect | Karyotype/CMA/WES |
|  | A345 | F | 26 | 25 | Bilateral duplicated kidneys | Karyotype/CMA |
|  | A921 | M | 26 | 28 | Bilateral duplicated kidneys | Karyotype/CMA |
|  | A014 | F | 34 | 26 | Right duplicated kidney, choroid plexus cysts | Karyotype/CMA |
|  | A451 | F | 26 | 26 | Right duplicated kidney, single umbilical artery | Karyotype/CMA |
|  | A547 | M | 26 | 24 | Left duplicated kidney, polyhydramnios | Karyotype/CMA |
|  | A548 | M | 30 | 23 | Right duplicated kidney | Karyotype/CMA |
|  | A066 | M | 29 | 24 | Right duplicated kidney | Karyotype/CMA |
|  | A880 | M | 29 | 24 | Left duplicated kidney | Karyotype/CMA |
|  | A565 | F | 36 | 23 | Left duplicated kidney | Karyotype/CMA |
|  | A735 | F | 33 | 23 | Bilateral duplicated kidneys, abnormal skeletal morphology | Karyotype/CMA |
|  | A738 | M | 29 | 22 | Left duplicated kidney, strong light spot in the left ventricle | Karyotype/CMA |
|  | A782 | F | 29 | 23 | Left duplicated kidney, choroid plexus cysts | Karyotype/CMA |
|  | A232 | M | 27 | 29 | Bilateral duplicated kidneys | CMA |
|  | A788 | M | 32 | 28 | Left duplicated kidney, polyhydramnios, short limbs | Karyotype/CMA |
|  | A877 | F | 31 | 27 | Bilateral duplicated kidneys | Karyotype/CMA |
|  | A989 | M | 37 | 30 | Right duplicated kidney | CMA |
|  | C0518 | F | 40 | 20 | Left duplicated kidney | Karyotype/CNV-seq |
|  | A869 | M | 32 | 24 | Bilateral duplicated kidneys, single umbilical artery | Karyotype/CMA |
|  | C1567 | F | 32 | 30 | Right duplicated kidney | CNV-seq |
|  | C1989 | M | 34 | 30 | Left duplicated kidney | CNV-seq |
|  | C2056 | F | 24 | 23 | Left duplicated kidney | Karyotype/CNV-seq |
|  | C2145 | M | 28 | 24 | Left duplicated kidney | Karyotype/CNV-seq |
|  | A495 | M | 33 | 23 | Right duplicated kidney | Karyotype/CMA |
|  | C0509 | M | 25 | 24 | Right duplicated kidney | Karyotype/CNV-seq |
|  | C0636 | F | 30 | 30 | Right duplicated collecting system, choroid plexus cysts | CNV-seq |
|  | A982 | M | 30 | 25 | Left duplicated collecting system | Karyotype/CMA |
| More than one renal anomaly | A023 | M | 27 | 22 | Left enlarged kidney, renal cyst | Karyotype/CMA/WES |
|  | A240 | F | 34 | 30 | Left duplicated kidney, hydronephrosis | CMA/WES |
|  | 6325 | M | 29 | 23 | Right polycystic kidney, left duplicated kidney | Karyotype/CNV-seq/WES |
|  | 1199 | F | 28 | 22 | Bilateral enlarged kidneys, hyperechogenic kidneys polydactyly, intrauterine growth retardation | Karyotype/CMA/WES |
|  | 0248 | M | 28 | 26 | Bilateral enlarged kidneys, hydronephrosis | Karyotype/CMA/WES |
|  | 0869 | M | 28 | 24 | Right enlarged kidney, pyelectasis, ureterectasia | Karyotype/CNV-seq/WES |
|  | 1230 | F | 31 | 19 | Left duplicated kidney, renal collection system separation, ureterectasia, fetal choroid plexus cysts | Karyotype/CNV-seq/WES |
|  | 1260 | M | 23 | 22 | Right hyperechogenic kidneys, right renal cyst | Karyotype/CNV-seq/WES |
|  | 0535 | M | 35 | 22 | Left pelvic kidney, renal dysplasia | Karyotype/CNV-seq/WES |
|  | 1688 | M | 28 | 24 | Bilateral enlarged kidneys, bilateral kidneys cortex hyperechogenic | Karyotype/CNV-seq/WES |
|  | 2110 | F | 18 | 26 | Left pelvic kidney, polycystic kidney | Karyotype/CNV-seq/WES |
|  | 2170 | M | 27 | 18 | Renal collection system separation, hyperechogenic kidneys, cystic hygroma, increased nuchal translucency | Karyotype/CNV-seq/WES |
|  | 2206 | F | 27 | 26 | Left duplicated kidney, hydronephrosis | Karyotype/CNV-seq/WES |
|  | 2290 | M | 30 | 24 | Right pelvic kidney, renal dysplasia | Karyotype/CNV-seq/WES |
|  | 2297 | M | 34 | 23 | Left pelvic kidney, renal dysplasia | Karyotype/CNV-seq/WES |
|  | 2003 | F | 32 | 25 | Left polycystic kidney, right hydronephrosis, hydronephrosis | Karyotype/CNV-seq/WES |
|  | 0003 | M | 36 | 18 | Renal dysplasia, ureterectasia | Karyotype/CNV-seq/WES |
|  | 0183 | M | 29 | 23 | Bilateral enlarged kidneys, hyperechogenic kidney | Karyotype/CNV-seq/WES |
|  | A410 | M | 33 | 24 | Duplicated kidney, ureterectasia | Karyotype/CMA |
|  | A873 | F | 24 | 24 | Duplicated kidney, ureterectasia | Karyotype/CMA |
|  | A033 | F | 24 | 24 | Right polycystic kidney, pyelectasis | Karyotype/CMA |
|  | A157 | M | 31 | 24 | Multicystic dysplastic kidney, right pelvic kidney | Karyotype/CMA |
|  | A784 | M | 24 | 24 | Multicystic dysplastic kidney, left pelvic kidney | Karyotype/CMA |
|  | A092 | F | 25 | 26 | Duplex kidney, ureterectasia | Karyotype/CMA |
|  | A096 | M | 31 | 25 | Right pelvic kidney, polycystic kidney | Karyotype/CMA |
|  | A673 | M | 31 | 24 | Hydronephrosis, right renal cyst | Karyotype/CMA |
|  | A210 | M | 32 | 29 | Hyperechogenic kidneys, renal dysplasia | CMA |
|  | A656 | F | 25 | 25 | Left polycystic kidney, ureterectasia | Karyotype/CMA |
|  | A147 | M | 21 | 22 | Hyperechogenic kidneys, pyelectasis, echogenic fetal bowel | Karyotype/CMA |
|  | A272 | M | 35 | 31 | Enlarged kidney, hyperechogenic kidneys | CMA |
|  | A351 | M | 28 | 23 | Pelvic kidney, multicystic dysplastic kidney | Karyotype/CMA |
|  | A722 | M | 29 | 23 | Pelvic kidney, renal dysplasia | Karyotype/CMA |
|  | A176 | M | 28 | 17 | Hyperechogenic kidneys, hydronephrosis, pyelectasis, ureterectasia, echogenic fetal bowel | Karyotype/CMA |
|  | A324 | M | 35 | 25 | Right polycystic kidney, ureterectasia | Karyotype/CMA |
|  | A804 | F | 40 | 30 | Duplicated kidney, hydronephrosis | Karyotype/CMA |
|  | 0955 | M | 30 | 25 | Hyperechogenic kidneys, hydronephrosis, strong light spot in the left ventricle | Karyotype/CNV-seq |
|  | A1203 | F | 33 | 24 | Left polycystic kidney, renal dysplasia, single umbilical artery | Karyotype/CMA |
|  | C1509 | F | 28 | 24 | Hyperechogenic kidneys, hydronephrosis, echogenic fetal bowel | Karyotype/CNV-seq |
|  | A1804 | M | 37 | 24 | Renal dysplasia, pelvic kidney, genital abnormality | Karyotype/CMA |
|  | C2205 | M | 30 | 20 | Hyperechogenic kidneys, polycystic kidney | Karyotype/CNV-seq |
|  | A2241 | M | 28 | 26 | Ureterectasia, renal cyst | Karyotype/CMA |
|  | A406 | F | 29 | 28 | Duplicated kidney, pyelectasis, ureterectasia | Karyotype/CMA |
|  | C0601 | M | 37 | 23 | Polycystic kidney, ureterectasia | Karyotype/CNV-seq |
|  | C0705 | F | 30 | 18 | Renal dysplasia, pelvic kidney | Karyotype/CNV-seq |

F, female; M, male; MA, maternal age; GA, gestational age; y, years; w, weeks. AMN, amniocentesis. Karyotyping was not performed in pregnancies after 28 weeks because amniotic cells were difficult to grow against the wall.
